# The 12-HHT/BLT2/NO Axis Is Associated to the Wound Healing and Skin Condition in Different Glycaemic States

**DOI:** 10.3390/medsci7040065

**Published:** 2019-04-24

**Authors:** Alberto Leguina-Ruzzi, Rina Ortiz Diban, Victoria Velarde

**Affiliations:** 1 Juntendo University, School of Medicine, Tokyo 113-8431, Japan; 2 Department of Mitochondrial Physiology, Institute of Physiology AS CR, v.v.l., 14220 Prague, Czech Republic; 3 Centro de Biotecnología, Universidad Técnica Federico Santa María, Avenida España 1680, Valparaíso 110-V, Chile; rortizdiban@gmail.com; 4 Departamento de Física, Universidad Técnica Federico Santa María, Avenida España 1680, Valparaíso 110-V, Chile; 5 Faculty of Biological Sciences, Department of Physiology, Pontifical Catholic University of Chile, Avda. Libertador Bernardo O’Higgins 340, Santiago 8331010, Chile; vvelarde@bio.puc.cl

**Keywords:** diabetes, BLT2, skin damage’ epithelial integrity, NO, 12-HHT

## Abstract

Type 2 diabetes affects over 340 million people worldwide. This condition can go unnoticed and undiagnosed for years, leading to a late stage where high glycaemia produces complications such as delayed wound healing. Studies have shown that 12-HHT through BLT2, accelerates keratinocyte migration and wound healing. Additionally, evidence has shown the role of nitric oxide as a pro-regenerative mediator, which is decreased in diabetes. Our main goal was to study the association between the 12-HHT/BLT2 axis and the nitric oxide production in wound healing under different glycaemia conditions. For that purpose, we used in vivo and in vitro models. Our results show that the skin from diabetic mice showed reduced BLT2 and iNOS mRNA, TEER, 12-HHT, nitrites, and tight junction levels, accompanied by higher MMP9 mRNA levels. Furthermore, a positive correlation between BLT2 mRNA and nitrites was observed. In vitro, HaCaT-BLT2 cells showed higher nitric oxide and tight junction levels, and reduced MMP9 mRNA levels, compared to mock-keratinocytes under low and high glucose condition. The wound healing capacity was associated with higher nitric oxide production and was affected by the NOS inhibition. We suggest that the BLT2 expression improves the keratinocyte response to hyperglycaemia, associated with the production of nitric oxide.

## 1. Introduction

Type 2 diabetes (T2D) is a metabolic disease that affects over 340 million people around the world [1]. This condition is a consequence of insulin resistance, in which cells fail to use insulin properly. It can go unnoticed and undiagnosed for years, passing through a normal glucose/high insulin state to a late stage where high blood sugar causes tissue damage as delayed wound healing caused by a chronic inflammatory and pro-oxidative state [2]. Wound healing in diabetes is impaired by factors that are associated with the physiological process traditionally divided into four phases: haemostasis, inflammation, proliferation, and maturation or remodelling. Hyperglycaemia can alter these four stages through direct mediators such as the advanced glycation end-products (AGEs) and alteration in intermediate metabolism [3]. 

Current research has shown that some eicosanoids decrease the insulin signalling [4]. However, 12-Hydroxyheptadecatrienoic acid (12-HHT) through its receptor Leukotriene B4 receptor type 2 (BLT2) accelerates keratinocyte migration and wound healing [5], which could be beneficial in a diabetic state. BLT2 is a G-Protein Coupled Receptor (GPCR) that binds to Leukotriene B4 (LTB4) and 12-HHT, and is mainly expressed in epithelial cells, such as epidermal keratinocytes and intestinal epithelial cells [6,7,8]. We previously reported that BLT2 activation improves epithelial integrity and function by regulating differentiation markers, cytokines and matrix metallopeptidase (MMP9), attenuating the damaging effects of high glucose levels, thereby accelerating wound healing [9]. Furthermore, the skin from diabetic mice presents higher levels of oxidative stress, lower levels of adhesion proteins and alterations in lipid mediators, particularly a reduction on 12-HHT [10].

Tight junctions (TJ) are proteins involved in proliferation and differentiation in keratinocytes. Claudin-4 and occludin have been reported as pivotal in the wound healing process [11]. 

Additionally, evidence has emerged about the role of nitric oxide (NO) as a pro-regenerative mediator in the skin [12], however no clear interconnection between eicosanoids and NO has been established. Importantly, we previously reported that NO and occludin decrease in a T2D mouse model [10]; however, there are no reports linking BLT2 and this free radical, as a mechanism associated to reepithelisation.

In this context, considering that the keratinocyte is the predominant cell type in the epidermis responsible for skin repair and is highly susceptible to damage, the study of these cells would present evidence about the participation of BLT2 in the physio pathological hyperglycaemic condition in T2D. Our hypothesis is that BLT2 in keratinocytes is a regenerative receptor that restores the tissue damage through NO production, offers protection from chronic inflammation and maintains the skin barrier integrity.

## 2. Materials and Methods

### 2.1. Animal Model of Type 2 Diabetes

C57BL/6J male mice (*n* = 12) of 15–21 weeks of age (average weight 23 ± 0.5 g) were divided into two groups. The first group was fed with a low fat (LF) chow D12450B as a control and the second was kept on a high fat (HF) diet D12492. The percentage of fat content in the diets were 10 kcal% fat and 60 kcal% fat, respectively. Mice were kept for 5 weeks in total on the diet. Glucose, insulin, lipids and weight were controlled weekly after 6 h of fasting. The study protocol (ID: ALR2015-2016) was approved by the Ethics Committee for Animal Experimentation at Juntendo University, Japan (2015–2016).

Triglycerides and blood glucose levels were measured using CardioChek^®^ PA (catalogue No. 0197) and the compatible PTS Panel^®^ test strips. Insulin was measured using the Ultrasensitive Mouse ELISA kit (Mercodia, Uppsala, Sweden, article No. 10-1249-01).

### 2.2. Transepithelial/Transendothelial Electrical Resistance Measurement

Ex vivo transepithelial/transendothelial electrical resistance (TEER) measurement was performed using a modification of the protocol described in the literature [13]. Skin samples with a diameter of 8 mm and a thickness of 1 mm were obtained from the back of the animal using disposable biopsy punches (Kai Medical, catalogue No. BP-80F). Then, they were placed onto a 12 mm polycarbonate filter with a 0.4 µm of pore diameter (Millicell Merck Millipore, Burlington, Massachusetts, USA, catalogue No. PIH01250) and suspended inside a cell culture well containing PBS. The epidermis was kept facing up. The TEER was measured immediately using the Millicell® ERS-2 Voltohmmeter (Millipore, Burlington, Massachusetts, USA, catalogue No. MERS00002).

### 2.3. 1-Hydroxyheptadecatrienoic Acid Quantification

For the determination of 12-HHT, the eicosanoid was extracted from skin with methanol containing deuterium-labelled internal standards. Each sample was diluted with water to yield a final methanol concentration of 20%, and then loaded on Oasis HLB cartridges (Waters). Eicosanoids in each sample were quantified by liquid chromatography–mass spectrometry (LC-MS/MS) using a Shimadzu liquid chromatography system and tandem-connected a TSQ Quantum Ultra triple quadrupole mass spectrometer equipped with an electrospray ionisation system (Thermo Fisher Scientific, Waltham, Massachusetts, USA). Each sample was analysed with an analytical column, a Capcell Pak C18 MGS3 (Shiseido, Tokyo, Japan). 

### 2.4. Western Blot

To determine relative levels of claudin-4, occludin and β-actin proteins, skin samples or cells were lysed in RIPA buffer (Tris-HCl 20 mM, NaCl 150 mM, Na_2_EDTA 1 mM, EGTA 1 mM, and NP-40 1%) containing protease inhibitors (1 mg/mL aminocaproic acid, 1 mg/mL benzamidine, 0.2 mg/mL SBTI and PMSF 3 mmol/L) and phosphatase inhibitors (0.012 mg/mL sodium orthovanadate, 4.46 mg/mL sodium pyrophosphate and 4.2 mg/mL sodium fluoride). Protein concentration was determined by the method of BCA. Proteins (30 µg) from lysates were separated by electrophoresis in 10% SDS-polyacrylamide gel (SDS-PAGE). Proteins were transferred to a 0.45 µm PVDF membrane, which was blocked with 5% non-fat milk and 1% BSA in PBS containing 0.05% Tween-20 at room temperature. Then, the PVDF membrane was incubated overnight at 4 °C with the primary antibody anti-occludin pAb (Thermo Fisher Scientific-Invitrogen catalogue No. 71–1500), claudin-4 pAb (Abcam catalogue No. Ab53156) or anti-β-actin mAb (Sigma–Aldrich catalogue No. A2228) at 1:500 of dilution, followed by incubation with secondary antibody conjugated to peroxidase at 1:1000 of dilution (Santa Cruz Biotechnology, Dallas, Texas) for 1 h at room temperature. Immunoreactive bands were visualised using a chemiluminescent reagent (Western Lightning, Perkin Elmer) according to the procedure described by the supplier. Chemiluminescence was detected by the Chemidoc-IT Imaging System (UVP, LLC) and immunoreactive bands were analysed by densitometry analysis using the ImageJ software (National Institutes of Health).

### 2.5. Cell Culture

Spontaneously transformed aneuploid immortal keratinocyte cell line (HaCaT) were transfected with a FLAG-tagged human BLT2-pCXN2.1 vector, or with the empty pCXN2.1 vector as a control. Stable transfection was achieved though the selection of cells with 1 mg/mL G418 (Wako Pure Chemical Industries, catalogue No. 071-06431) in culture medium and incubated with an anti-FLAG antibody (2H8), followed by an Alexa Fluor 488-conjugated goat anti-mouse IgG secondary antibody (Life Technologies, Carlsbad, CA, USA, A-11001). HaCaT cells were maintained in D-MEM (Wako Pure Chemical Industries, Tokyo, Japan, catalogue No. 044–29765) containing 10% foetal calf serum (FCS) Gibco® (Thermo Fisher Scientific, Waltham, Massachusetts, USA, catalogue No. 16000–069). For the normal glucose treatment, D-MEM containing 5 mM glucose (Wako Pure Chemical Industries Tokyo, Japan, catalogue No. 041–29775) was used. For the high glucose treatment, keratinocytes were maintained in D-MEM 25 mM glucose (Wako Pure Chemical Industries, Tokyo, Japan, 044–29765) and in both conditions the cells were treated without FBS, for 48 h.

### 2.6. 2′,7′-dichlorodihydrofluorescein diacetate (DCF-2DA) Assay

Cells (1 × 10^4^) were seeded in the black clear bottom of the 96-well dish for 48 h. Then, they were washed once with warm 1× PBS and maintained at 37 °C with 20 µmol/L DCF-2DA (GORYO Chemical, Sapporo Japan) for 1 h. After the incubation, the cells were washed twice and 100 µL of phenol red free medium with the proper glucose concentrations was deposited in each well. Immediately and every 2 h, the fluorescence (ex. 495, em. 515) and phase contrast measurements were made using the IncuCyte zoom software (Essen BioScience), for 2 days in total. L-NAME (100 µM) and NONOate (100 µM) were used as negative and positive control, respectively.

### 2.7. Nitrites Colorimetric Measurement

Nitrites were measured using the Griess reaction. Briefly, cells (1 × 10^5^) were seeded in 6-well dishes for 24 h; then, the medium was replaced by phenol red free-DMEM with the specified glucose concentrations. Every 12 h, starting at the moment of medium change, 300 µL of supernatant were taken followed by a 300× *g* centrifugation for 5 min. For skin, the tissue was homogenised in 2 mL of 1× phosphate buffer/saline (PBS) using gentle MACS Dissociators. After that, the samples were centrifuged at 1500× *g* for 5 min followed by a deproteinisation at 11,200× *g* for 15 min using 30 kDa pore Millipore Centrifugal Filter Unites. The performance was checked measuring the protein concentration before and after the procedure, and a 20-times reduction in the protein levels was considered as appropriate for the assay.

Supernatant (150 µL) or deproteinised skin homogenised samples were incubated for 30 min at room temperature with Griess Reagent Kit (ThermoFisher Scientific). The colorimetric reaction was measured in an endpoint at 595 nm. The values were interpolated using a nitrites standard curve and for skin, the measurement was normalised by micrograms of tissue.

### 2.8. In Vitro Wound Healing Assay

HaCaT cells (1.5 × 10^4^ cells/well) were seeded onto a collagen I-coated 96-well plate (Essen BioScience, catalogue No. 4379) and incubated in a standard CO2 incubator for 48 h to form a cell monolayer, before being treated with 2 µg/mL Mitomycin C (Sigma-Aldrich, catalogue No. M0503) for 2 h. Wounds were made with the 96-well WoundMaker™ (Essen BioScience, 4493). To remove any detached cells, wounded cell layers were washed twice with culture medium and then incubated with 100 µL of medium containing the appropriate glucose concentrations. Images of the wounds were automatically acquired within the CO_2_ incubator using the IncuCyte™ ZOOM software package (Essen BioScience, Ann Arbor, Michigan, USA, catalogue No. 2016A). Typical kinetic updates were taken at 3 h intervals for the duration of the experiment (48 h). Finally, cell wound closure analysis was performed using the IncuCyte™ ZOOM software.

### 2.9. Reverse Transcription and Quantitative PCR

Total RNA was extracted from cells by the acid guanidinium thiocyanate-phenol-chloroform extraction method using Trizol reagent (15596-018; Life Technologies). Real-time polymerase chain reaction was performed FastStart Essential DNA Green Master Mix (Roche). The PCR amplification was carried out with initial denaturation at 95 °C for 20 s, followed by 45 cycles of 95 °C for 3 s and 60 °C for 30 s in a LightCycler 96 (Roche). The primers used were:

Murine MMP9 (Mmp9)
FW 5′-CCTACTCTGCCTGCACCACTAAA-3′
RV 5′-CTGCTTGCCCAGGAAGACGAA-3′
Human MMP9 (MMP9)
FW 5′-GACGCAGACATCGTCATCCAGTTT-3′
RV 5′-GCCGCGCCATCTGCGTTT-3′
Murine iNOS (Nos2)
FW 5′- CGAAACGCTTCACTTCCAA -3′
RV 5′- TGAGCCTATATTGCTGTGGCT -3′
Human iNOS (NOS2)
FW 5′-5′-CAGCGGGATGACTTTCCAA-3′
RV 5′-AGGCAAGATTTGGACCTGCA-3′
Murine nNOS (Nos1)
FW 5′- CTCACCCCGTCCTTTGAGTA-3′
RV 5′- GGTCGCTTTGACTCTCTTGG-3′
Human nNOS (NOS1)
FW 5′- TCT CCT CCT ACT CTG ACT CC-3′
RV 5′- TTG TGG ACA TTG GAT AGA CC-3′
Murine BLT2) (Blt2)
FW 5′-ACAGCCTTGGCTTTCTTCAG-3′
RV 5′-TGCCCCATTACTTTCAGCTT-3′
Murine β -actin (actb)
FW 5′-CATCCGTAAAGACCTCTATGCCAAC-3′
RV 5′-ATGGAGCCACCGATCCACA-3′
Human β-actin (ACTB)
FW 5′-TGGCACCCAGCACAATGAA-3′
RV 5′-CTAAGTCATAGTCCGCCTAGAAGCA-3′

Gene expression levels were calculated using the 2^−ΔΔCt^ method after normalisation to the expression level of the standard housekeeping gene, β-actin.

### 2.10. Statistical Analysis

For all values, mean and standard error of the mean (SEM) were calculated. Results are presented as mean ± SEM. Statistical analyses were performed using an unpaired Student’s *t*-test (when comparing two groups), one-way non-parametric ANOVA (Kruskal–Wallis test) followed by Bonferroni post hoc analysis (for the comparison of more than two groups), or correlation analysis. Statistical significance was set at *p* < 0.05. All statistics were calculated using Prism (Graphpad Software).

## 3. Results

### 3.1. Skin Condition in T2D Animal Model

Biochemical and physical evaluation showed that five weeks of HF diet produced an exacerbated weight increase, and augmented plasmatic levels of basal insulin and triglycerides compared to mice under control diet (Table 1). These characteristics are classic features of the early stages of T2D (insulin resistance).

**Figure 1:**
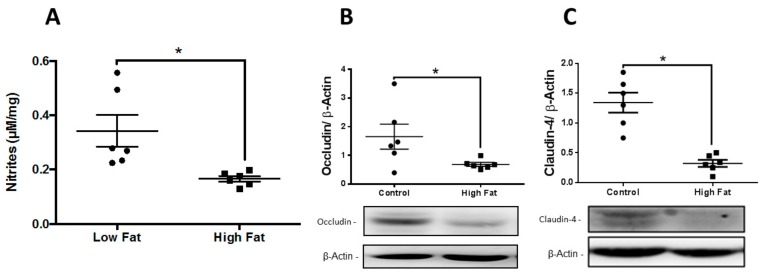
NO and occludin reduction on type 2 diabetic: Skin evaluation of NO levels (**A**); Western blot for occludin (**B**); and Western blot for claudin-4 (**C**). Data represent the mean ± SEM, *n* = 6 per group, * *p* < 0.05, Student-*t* test.

Additionally, the skin of diabetic mice presented lower TEER, 12-HHT and iNOS mRNA levels. Moreover, higher MMP9 mRNA amounts were observed (Table 1), accompanied with lower nitrites (Figure 1A), occludin (Figure 1B) and claudin-4 (Figure 1C) levels.

**Figure 2:**
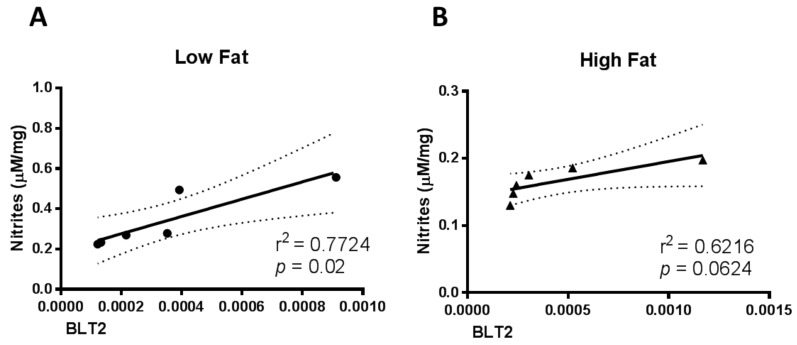
The NO levels positively correlate with BLT2 expression levels in low fat diet mice. Correlation between nitrites and BLT2 mRNA levels in skin from; (**A**) low fat diet mice; and (**B**) high fat diet mice. *n* = 6 per group.

Interestingly, under normal diet, there was a positive correlation between BLT2 protein and nitrite amounts in skin (Figure 2A). On the other hand, the tissue from HF diet fed mice presented a tendency on the positive correlation, suggesting a misbalance on the physiological metabolism (Figure 2B). It is important to highlight that more cause–effect evidence is required to confirm the observations.

### 3.2. In Vitro Effect of High Glucose

**Figure 3:**
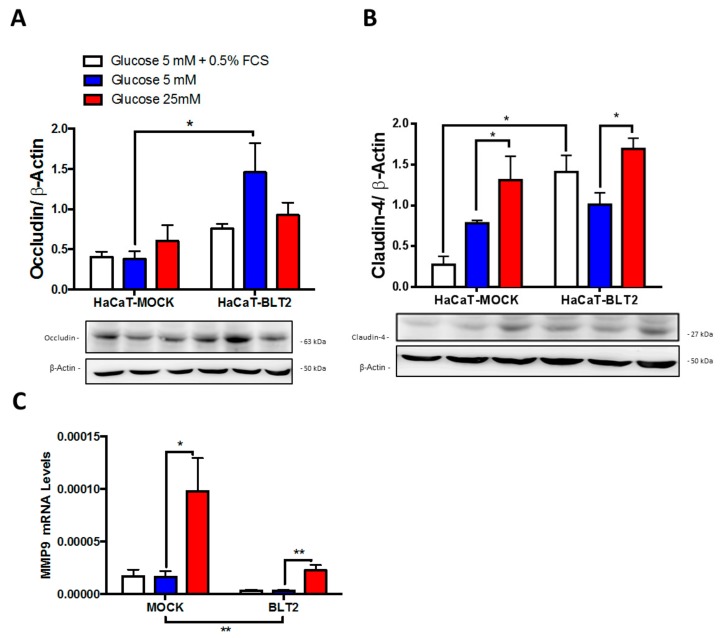
HaCaT-BLT2 cells present higher occludin levels and lower MMP9 expression: (**A**) Western blots for occludin; (**B**) Western blots for claudin-4; and (**C**) Q-PCR for MMP9 in different glucose levels for 48 h. Data represent the mean ± SEM of n = 3 independent experiments * *p* < 0.05, one-way non-parametric ANOVA (Kruskal–Wallis test).

In vitro, HaCaT-BLT2 cells grown in 5 mM glucose produced higher protein levels of occludin; in opposition, 25 mM glucose increased claudin-4. However, lower MMP9 mRNA amounts were observed in BLT2 expressing cells, compared with mock cells, an effect that was maintained after 48 h of glucose 25 mM (Figure 3A,B). Importantly, the condition 5 mM + 0.5% FCS included ~0.75 nM of 12-HHT and demonstrated that the presence of the activation of the GPCR induced increases in TJs and the effect of high glucose was not directly mediated by the receptors activity.

**Figure 4:**
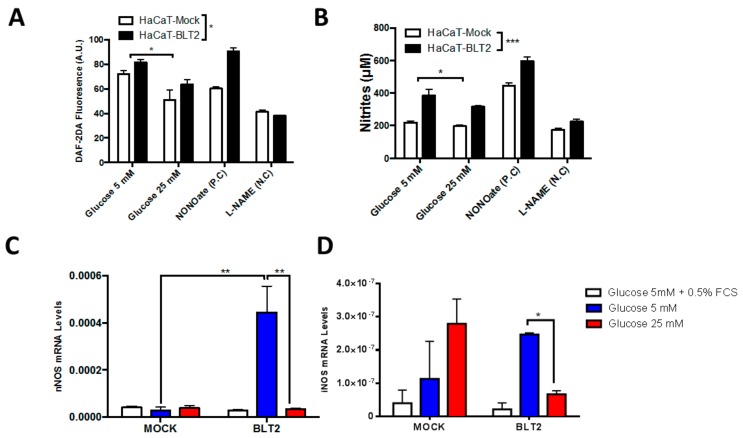
HaCaT-BLT2 cells exhibit higher NO production and nNOS expression levels. Nitric oxide measurement by: (**A**) DAF-2DA; and (**B**) Griess reaction after 48 h of low or high glucose levels. The 100 µM NONOate and 100 µM L-NAME were used as positive and negative controls, respectively. (**C**) Q-PCR for nNOS; and (**D**) Q-PCR for iNOS after 48 h. Data represent mean ± SEM, of *n* = 3 independent experiments. * *p* < 0.05, ** p < 0.01, *** *p* < 0.005, one-way non-parametric ANOVA (Kruskal–Wallis test).

Furthermore, BLT2 expressing keratinocytes produced higher amount of NO associated with higher nNOS mRNA levels, compared to HaCaT-MOCK cells in 5 mM glucose (Figure 4A–D). Importantly, the high glucose incubation for 48 h reduced the NO production, an effect partially avoided in BLT2 cells. However, only these cells exhibited a reduction in nNOS and iNOS in response a 25 mM glucose (Figure 4C,D). NONOate was used as positive control (PC) and L-NAME as negative control (NC), with an inexplicable differential effect, particularly for NONOate in BLT2 expressing cells.

### 3.3. In Vitro Effect of NO Inhibition or Donation on Wound Healing

**Figure 5:**
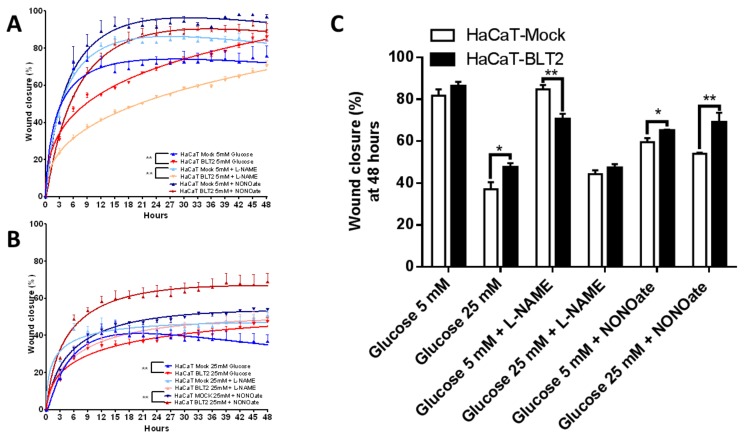
The inhibition of NOS or NO donation affects selectively to the BLT2 expressing cells. Wound healing assay, using the unspecific NOS inhibitor L-NAME at 100 µM or a NO donor at 100 µM for 48 h. (**A**) Quantification under low glucose (5 mM); or (**B**) quantification under high glucose (25 mM) for two days with measurements every 3 h. (**C**) Wound healing percentage after 48 h. Data represent mean ± SEM, of n = 3 independent experiments. * *p* < 0.05, ** *p* < 0.01, one-way non-parametric ANOVA (Kruskal–Wallis test).

As we previously described, high glucose reduced the wound healing capacity, an effect prevented by the BLT2 expression in a HaCaT [9]. The NOS unspecific inhibition by L-NAME at 5 mM glucose altered differentially the HaCaT-BLT2, reducing the wound closure. In opposition, the NO donation with NONOate at this glucose concentration increased the wound closure in both cell lines as expected, but a slight increase was observed particularly in the BLT2 expressing keratinocytes (Figure 5A). On the other hand, at 25 mM, L-NAME did not further reduce the wound healing, suggesting that the cells already reached the minimum migratory capacity; however, at high glucose, NONOate had a specific and inexplicable effect only in the cells that express this GPCR (Figure 5B). As shown in Figure 5C, after 48 h, the alterations were recognisable.

## 4. Discussion

In this study, we demonstrated using in vivo and in vitro strategies that high glucose induced a reduction in NO production and NOS downregulation in skin and keratinocytes. These changes in skin correlated with reduction in BLT2 expression and with impaired wound closure in cell cultures.

The use of a non-genetic, induced, murine T2D model represents an important novelty in the context of the results obtained in this study. Various reports have validated the use of a HF diet over several weeks to provide a model of T2D [14,15,16]. As we previously demonstrated, the diabetic mice exhibited important skin alterations and BLT2 expression in keratinocytes protected from the damage caused by hyperglycaemia [9]. 

Clinical and in vivo studies have associated the overexpression of MMP9 with photoaging [17], delayed wound healing [18] and infective lesions [19]. Moreover, the reduction in tight junctions composed of occludin and claudin-4 [20] and the reduction in TEER [21] reflected a lower skin barrier function and integrity. Our results show an altered skin in response to type 2 diabetes induced by a HF diet in vivo, which correlated with the hyperglycaemic state in vitro. 

It has been demonstrated that the 12-HHT/BLT2 axis enhances epithelial barrier function, accelerating wound healing and regulating the inflammatory response and MMPs production [5]. Interestingly, we report that the diabetic mice presented a reduction in skin 12-HHT without significant changes in BLT2; furthermore, the lack of this receptor in keratinocyte reduced the migration capacity, TEER and tight junctions. We previously used a knock out mice, in which we proved that the presence of BLT2 improved epithelial integrity and function by regulating differentiation markers and cytokines. Similarly, BLT2 attenuated the damaging effects of high glucose levels, thereby enhancing wound healing [10].

NO, a classic free radical with vasodilatation capacity [22], plays an important role in the maintenance and regulation of the skin and the integrity of its environment [23]. The keratinocytes, which make up the bulk of the epidermis, constitutively express the neuronal isoform of NO synthase (NOS1) and, under certain conditions, virtually all skin cells appear to be capable of expressing the inducible NOS isoform (NOS2) [24,25,26]. 

Since the discovery of the NO production in the skin, it has been stablished that it is an effective therapeutic factor for chronic wound healing as it is a potent anti-biofilm agent and plays a key role in active wound regeneration and new technical strategies have been explored for its therapeutic use [27,28,29,30]. 

In accordance with the previously reported evidence, we showed that diabetic mice induced by HF diet presented lower skin NO with iNOS downregulation and this was positively correlated with the BLT2 expression. More importantly, in vitro, we evidenced that BLT2 expressing keratinocytes produced higher NO levels, the reduction induced by high glucose was lower, and they responded better to NONOate and to L-NAME. In contrast to the in vivo results, this was related to the nNOS expression at low glucose (5 mM) and to iNOS only in response to 25 mM glucose for 48 h.

To our knowledge, this is the first report that evidences the direct relation between BLT2 and NO in association with wound healing without the use of the GPCR agonist; a previous report using HUVEC cells showed that LTB4 increases the nitrite production [31]. Moreover, in our in vivo results, we report a reduction in 12-HHT, the endogenous BLT2 agonist and in NO, accompanied by an impaired skin condition.

Although more experiments are needed to understand the mechanism underlying the 12-HHT/ BLT2/NO axis in keratinocytes, our results complement previous reports and open the possibilities for future clinical studies on wound healing management modulating the NO production through this pathway.

## 5. Conclusions

**Figure 6:**
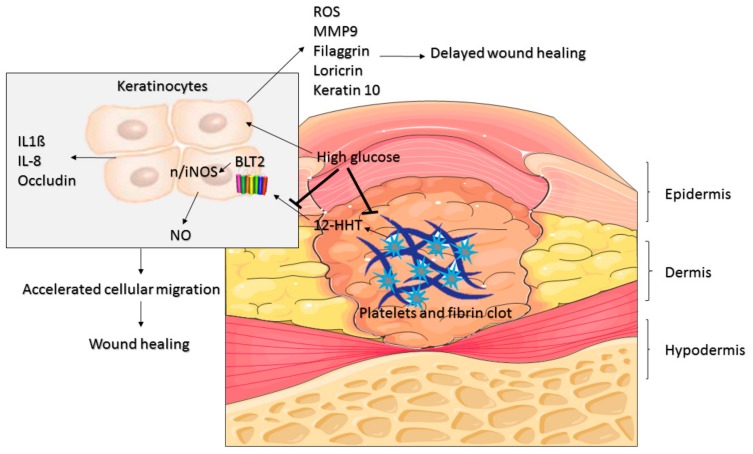
Proposed model of 12-HHT/BLT2/NO action in wound healing.

Based on our previous and current findings, we propose that 12-HHT via BLT2 induces an increase in NO, cytokines and tight junctions producing an accelerated keratinocytes migration. On the other hand, high glucose would inhibit this signalling and the local production of 12-HHT, generating an increase of oxidative stress, MMP9 and terminal differentiation markers, abrogating the wound healing capacity (Figure 6). It is pivotal to establish further in vivo studies to evaluate the association of this pathway and wound healing, as one of the main weakness of this study.

Mediators released by the keratinocyte accelerated the cellular migration and the inhibitory effect of high glucose.

It has been shown that insulin resistance and in particular a NO depletion can start from an early age [32], thereby increasing the predisposition for complications associated with diabetes in the later years of life. The clinical management of the complications of T2D is therefore an important public health issue; more work on this topic is required in the future.

## Figures and Tables

**Table 1:** General characteristics of the type 2 diabetic mice model

Physical and Biochemical Characteristics		
	Low Fat	High Fat
Weight (g)	26.88 ± 0.44	34.35 ± 0.61 **
Glucose (mg/dL)	111.7 ± 2.21	225 ± 7.13 **
Insulin (µg/L)	1.41 ± 0.10	8.04 ± 2.38 *
Triglycerides (mg/dL)	156.3 ± 2.23	192 ± 6.63 **
**Skin Characteristics**		
TEER (Ωcm^2^)	23.70 ± 1.30	11.30 ± 2.37 **
12-HHT (pg/mg)	15.22 ± 6.11	6.73 ± 2.35 *
iNOS mRNA (A.U)	4.47 × 10^−6^ ± 6.58 × 10^−7^	0.48 × 10^−6^ ± 2.68 × 10^−7^ **
nNOS mRNA (A.U)	4.8 × 10^−4^ ± 1.3 × 10^−4^	5.2 × 10^−4^ ± 1.5 × 10^−4^
MMP9 mRNA (A.U)	0.002 ± 0.0009	0.013 ± 0.0025 **
BLT2 mRNA (A.U)	3.5 × 10^−4^ ± 1.2 × 10^−4^	4.4 × 10^−4^ ± 1.5 × 10^−4^

Physical, biochemical and skin characteristics after five weeks of diet treatment. Data expressed as average ± SEM, *n* = 6 per group, * *p* < 0.05, ** *p* < 0.01 Student’s *t*-test.
